# Adoptive T Cell Therapy Following Haploidentical Hematopoietic Stem Cell Transplantation

**DOI:** 10.3389/fimmu.2019.01854

**Published:** 2019-08-06

**Authors:** Ping Zhang, Siok-Keen Tey

**Affiliations:** ^1^Clinical Translational Immunotherapy Laboratory, QIMR Berghofer Medical Research Institute, Herston, QLD, Australia; ^2^Department of Haematology and Bone Marrow Transplantation, Royal Brisbane and Women's Hospital, Herston, QLD, Australia; ^3^Faculty of Medicine, The University of Queensland, Brisbane, QLD, Australia

**Keywords:** haploidentical transplant, T cell immune reconstitution, adoptive T cell therapy, antigen-specific T cells, safety switch, gene modification

## Abstract

Delayed immune reconstitution and the consequently high rates of leukemia relapse and infectious complications are the main limitations of haploidentical hematopoietic stem cell transplantation. Donor T cell addback can accelerate immune reconstitution but the therapeutic window between graft-vs.-host disease and protective immunity is very narrow in the haploidentical transplant setting. Hence, strategies to improve the safety and efficacy of adoptive T cell transfer are particularly relevant in this setting. Adoptive T cell transfer strategies in haploidentical transplantation include the use of antigen-specific T cells, allodepletion and alloanergy induction, immune modulation by the co-infusion of regulatory cell populations, and the use of safety switch gene-modified T cells. Whilst common principles apply, there are features that are unique to haploidentical transplantation, where HLA-mismatching directly impacts on immune reconstitution, and shared vs. non-shared HLA-allele can be an important consideration in antigen-specific T cell therapy. This review will also present an update on safety switch gene-modified T cells, which can be conditionally deleted in the event of severe graft- vs.-host disease or other adverse events. Herpes Virus Simplex Thymidine Kinase (HSVtk) and inducible caspase-9 (iCasp9) are safety switches that have undergone multicenter studies in haploidentical transplantation with encouraging results. These gene-modified cells, which are trackable long-term, have also provided important insights on the fate of adoptively transferred T cells. In this review, we will discuss the biology of post-transplant T cell immune reconstitution and the impact of HLA-mismatching, and the different cellular therapy strategies that can help accelerate T cell immune reconstitution after haploidentical transplantation.

## Introduction

The past decade has seen a sharp increase in the number of haploidentical hematopoietic stem cell transplants (HSCT), which is driven by smaller family sizes and increased transplant activity amongst patients of non-European ancestries that are not well-represented in volunteer donor registries (1, 2). At the same time, outcomes of haploidentical HSCT have steadily improved, with some specialized centers reporting outcomes that are comparable to those of matched sibling and matched unrelated transplants (3–8). This remarkable progress can be attributed to advances in graft-engineering and critical refinements in conditioning regimen and immunosuppressive regimen, which together overcome the key barriers of graft rejection and lethal graft- vs.-host disease (GVHD). Three major haploidentical HSCT approaches have emerged: (1) intensive myeloablative conditioning regimen combined with *in vivo* T cell depletion with anti-thymocyte globulin (ATG) to enable the engraftment of megadose CD34-selected T cell depleted graft, which was pioneered in Perugia, Italy (9); (2) non-myeloablative or reduced-intensity conditioning followed by the infusion of unmanipulated T cell replete bone marrow or peripheral blood stem cell graft, followed by the depletion of alloreactive T cells *in vivo* with high-dose post-transplant cyclophosphamide (PTCy), which was pioneered in Baltimore, USA (10); and (3) high-intensity myeloablative conditioning regimen that incorporates ATG-based *in vivo* T cell depletion and intensive immunosuppression followed by the infusion of granulocyte colony-stimulating factor (G-CSF)-primed bone marrow or peripheral blood stem cell grafts, which was pioneered in Beijing, China (11). Despite the promising outcomes, infectious complications and relapse of underlying malignancies remain significant sources of transplant failure, especially following *ex vivo* T cell deplete haploidentical HSCT, where T cell immune reconstitution is particularly delayed. T cell reconstitution is numerically more rapid after T cell replete haploidentical HSCT using either PTCy or the Beijing approach (12–14), but the qualitative immune dysfunction that characterizes all forms of allogeneic HSCT is exacerbated by HLA-disparity in the haploidentical setting.

Adoptive T cell transfer has an established role in allogeneic HSCT and are particularly relevant in the haploidentical setting, where immune reconstitution is poorer and the immediate and near-universal availability of related donors provide added opportunities for advanced graft engineering and cellular therapy. The principles of adoptive T cell transfer after HLA-matched transplantation is broadly applicable to other transplant settings but the risk of GVHD, at least from donor-derived T cell therapy, is higher in the presence of HLA-mismatch, especially in haploidentical HSCT, where the precursor frequency of alloreactive T cells can be orders of magnitude higher (15). This lower therapeutic index has inspired new approaches, including the use of safety-switch modified T cells that can be conditionally deleted in the event of severe GVHD (16), and immune-modulatory approaches, such as the co-infusion of regulatory T cells (Tregs) together with conventional T cells (Tcons) (17), and allospecific T cell depletion and anergy induction (18).

In this manuscript, we will briefly review the features of immune reconstitution after haploidentical HCST, followed by detailed discussions on the use of adoptive T cell transfer, including an update on safety-switch gene-modified T cell addback.

## T Cell Reconstitution Following Haploidentical HSCT

The pattern and tempo of immune reconstitution is influenced by the specific transplant technique. In all cases, innate immunity reconstitutes faster, with natural killer (NK) cells and γδ-T cell reaching normal numbers within the first few weeks post-transplant (19). The reconstitution of adaptive immunity, both cellular and humoral, is significantly slower (20). T cells, which are key mediators of both GVHD and graft- vs.-leukemia effect, reconstitute via two distinct pathways: the expansion of T cells that are contained within the stem cell graft, and the development of new thymic emigrants from donor hematopoietic stem cells (20, 21). The lymphopenic environment created by pre-transplant conditioning promotes cytokine-driven expansion of T cells within the graft. Subsequent antigen exposure, including viral antigens, provides further expansion of antigen-specific T cells (14, 22). In T cell deplete transplants where there are only small numbers of contaminating T cells, these early reconstituting T cells have a narrow T cell receptor (TCR) repertoire. In one study, 80% of the T cells at 2 months post-transplant could be accounted by as few as 13–504 TCR clonotypes, with overlaps found with T cells in the graft (23). In haploidentical HSCT with PTCy, the number of T cells infused is large, but a significant proportion is subsequently deleted by cyclophosphamide. Although T cell count recovery is much more rapid, the T cells are predominantly CD45RA(-)CCR7(-) effector memory and CD45(+)CCR7(-) terminally differentiated TEMRA in phenotype, and have a lower TCR repertoire diversity that is not fully restored even at 1 year post-transplant (14). The impact of transplantation platform on TCR repertoire reconstitution is difficult to quantify because of differences in baseline patient characteristics and TCR sequencing technologies, but delayed reconstitution of TCR repertoire is common to all allogeneic HSCT, including fully HLA-matched transplantation (24), and can have important implications on functional immune reconstitution.

Restoration of TCR repertoire through the export of naïve T cells from the thymus is a slow process which takes 1–2 years and is affected by recipient age and GVHD (25–27). Thymic T cell selection is determined by the affinity of TCR to peptide-MHC expressed in the thymic microenvironment, specifically by the thymus epithelial cells (TECs) and bone-marrow-derived antigen-presenting cells (28). T cell selection comprises two sequential stages: positive selection which prevents death by neglect of double positive thymocytes that express TCR of intermediate affinity for self-peptide MHC on cortical TECs; followed by negative selection, during which thymocytes with high-affinity to self-peptide MHC are deleted by apoptosis. Negative selection occurs predominantly in the thymic medulla and is mediated by a broader range of cell types, including medullary TECs and a range of bone marrow-derived cells: resident and migratory conventional dendritic cells, plasmacytoid dendritic cells and B cells (28, 29). Following allogeneic HSCT, the hematopoietic antigen presenting cells are of donor origin, whereas the TECs remain of recipient origin. In haploidentical HSCT, there is a degree of mismatch between the MHC that effect thymic selection and the MHC that is expressed on peripheral tissue. In addition, the thymus is a target organ for GVHD, and the thymic stroma can also be damaged by the conditioning regimen. The resulting thymic dysfunction impairs thymic export of naïve T cells and disruption to negative selection allows escape of autoreactive T cells to the periphery, which exacerbates GVHD and leads to further thymic dysfunction (30, 31). The recovery of thymic export after allogeneic HSCT also disproportionately affects Tregs, with lower proportions of recent thymic emigrants within the Treg compartment as compared to CD4 and CD8 conventional T cell compartments, and this imbalance further contributes to post-transplant immune dysregulation (32).

Cytomegalovirus (CMV) is a strong driver of early T cell reconstitution, especially in the CD8 effector memory and TEMRA compartments (14, 22). Interestingly, CMV also promotes a convergence of TCR repertoire between recipients and donors, likely because CMV-specific T cells can constitute a significant fraction of the T cell compartment (14). It is important to note here that HLA-mismatching in itself has an impact on functional immune reconstitution: a proportion of pathogen-specific memory T cells transferred within the graft will bear TCRs that are restricted to non-shared HLA alleles, which are not expressed by pathogen-infected recipient non-hematopoietic cells. Conversely, there is a deficiency of T cells that can bind recipient HLA alleles that are not present in the donor. The net effect is a functional defect in the TCR repertoire, which will take months and years to recover, with the reconstitution of new thymic emigrants.

## High Frequency of MHC-alloreactive T Cells as a Limitation to Unmanipulated T Cell Addback

The delayed addback of defined doses of T cells after T cell deplete transplantation can potentially accelerate T cell reconstitution without excessive risks of life-threatening GVHD. Intensive pre-transplant conditioning induces tissue damage, and the resulting cytokine storm activates recipient antigen-presenting cells and enhances the priming and Th1/Th17 polarization of donor alloreactive T cells (33, 34). Hence, donor T cell infusion after the resolution of cytokine storm should in theory be associated with lower risks of severe GVHD. However, in the presence of HLA-disparity, the risk of GVHD is high even with small T cell doses. In early studies using T cell depletion by soybean agglutination and E-rosetting, fatal acute GVHD occurred with T cell graft contamination of 1 × 10^6^/kg despite concurrent administration of ATG (35). Indeed, the actual safe dose of T cell would turn out to be much lower.

The precursor frequency of alloreactive T cells in HLA-mismatched transplantation is estimated at 1–10% based on *in vitro* and *in vivo* functional assays (15, 36). The molecular basis for this vast repertoire of MHC-alloreactive T cells is not fully elucidated. Current evidence suggests that TCRs that are positively selected on low and intermediate affinity interactions with self MHC/peptide can sometimes cross-react with allogeneic MHC/peptide because there is a degree of flexibility in TCR-MHC/peptide interaction. These allogeneic MHC/peptide interactions can be of high affinity because the mismatched recipient MHC is not expressed in the donor's thymus and, hence, physiological deletion of high affinity TCR has not occurred (15). Cross-reactivity can occur by molecular mimicry: for example, a single TCR that is specific to HLA-B^*^0801 presenting FLRGRAYGL peptide from an Epstein-Barr virus (EBV) nuclear antigen can also recognize HLA-B^*^3501 presenting KPIVVLHGY peptide from human cytochrome P450 because of structural homology in the regions implicated in TCR recognition “hot spots” (37, 38). In other cases, cross-reactivity occurs without a need for molecular mimicry: a single TCR can recognize self- and allogeneic-MHC/peptide complexes through unique amino acid contacts, which results in divergent binding orientations (36). The relative contribution of the different molecular mechanisms is yet to be defined but it is clear that the magnitude of allogeneic MHC cross-reactive T cells can be very large: in one study, 45% of virus-specific T cell clones were found to cross-react with allogeneic HLA molecules *in vitro* (39). Thus, even small doses of T cells has the potential to cause life-threatening GVHD in haploidentical transplantation.

G-CSF priming is perhaps one of the earliest, albeit unintended, form of T cell immune modulation. The incidence of acute GVHD after transplantation with G-CSF mobilized peripheral blood stem cell grafts is comparable to that after bone marrow grafts despite the former having 10-fold higher number of T cells (40). This has been attributed to Th2 polarization (41), promotion of regulatory T cells (42) and expansion of regulatory antigen-presenting cells (43), which collectively contribute to the lower rate of acute GVHD, although at the expense of increased Th17 polarization and chronic GVHD (44, 45). Despite this, the safe dose of G-CSF-primed donor lymphocyte infusion (DLI) is very low. In a dose-finding study, adult patients undergoing CD34-selected haploidentical HSCT without post-transplant immunosuppression received prophylactic DLI from day +28 onwards, using the CD34-negative fraction of the graft. A dose of 3 × 10^4^ CD3^+^ T cells/kg induced grade II acute GVHD in 2 out of 2 patients. Three lots of monthly DLI at 1 × 10^4^ CD3/kg/dose was found to be safe but incremental DLI to 3 × 10^4^ CD3^+^ T cells/kg again induced high rates of acute GVHD (Table 1) (46). CD4 immune reconstitution remained very delayed despite DLI, relapse rate was high in patients without GVHD, and two patients in this small study later died from GVHD. A similar approach was studied in the pediatric population with G-CSF-primed DLI at 4–6 weeks after T cell deplete transplant. In this study, weekly methotrexate was administered post-DLI to limit the risk of acute GVHD. It was found that modest T cell doses at 3–5 × 10^4^ cells/kg could achieve their target endpoint of at least 67% of children reaching CD4 T cell count >100/uL by day +120 post-transplant, nearly all of which were memory T cells, suggesting that they originated form the DLI fraction. Grade II–IV acute GVHD occurred in seven out of 35 children (49). In the T cell replete setting, investigators at Beijing used cryopreserved excess peripheral blood stem cell grafts for subsequent DLI, either in response to disease relapse or prophylactically in high-risk patients. In one study, 20 patients with leukemia relapse received G-CSF primed DLI at a dose of 0.07–4.4 × 10^8^ CD3^+^ T cells/kg. Grade III-IV acute GVHD occurred in 5 out of 9 patients who did not receive GVHD prophylaxis and in 1 out of 11 patients who received post-DLI cyclosporine or methotrexate; with overall disease response rate of 70% (47). In a recent study from the same group, 31 patients who underwent haploidentical HSCT for high-risk leukemia received prophylactic DLI at a median of 77 days post-transplant, at a median dose of 1.8 × 10^7^ CD3^+^ T cells/kg. Grade III–IV acute GVHD occurred in only 10% of patients, but a significant proportion were on prophylactic cyclosporine (48). Together, these studies demonstrate that the safe dose of G-CSF-primed DLI without concurrent immunosuppression is in the range of 1–3 × 10^4^ CD3^+^ T cells/kg; higher doses will require concurrent immunosuppression, which may limit their efficacy.

**Table 1:** Selected studies using G-CSF primed peripheral blood mononuclear cell (G-PBMC) add-back following haploidentical HSCT.

**Underlying disease**	**Transplant protocol**	**Study cohort**	**T cell dose/time of infusion**	**GVHD prophylaxis after G-PBMC**	**Outcome**	**References**
AML, ALL, CML, MDS, NHL	CD34+ selected stem cell graft, with ATG and pre-transplant CSA and steroids. No post-transplant immunosuppression.	Adults (*n* = 12) but only *n* = 11 received DLI	3 × 10^4^/kg (*n* = 2), 1 × 10^4^/kg/month × 3 doses, or 1, 3, 10 × 10^4^/kg/month or 1–5 × 10^5^/kg every 2 weeks (*n* = 9) (therapeutic DLI after relapse)	None	aGVHD in 2/2 at 3 × 10^4^/kg; aGVHD grade I not requiring systemic treatment at 1 × 10^4^/kg/month but high relapse rate; GVHD in all patients with dose-escalated DLI or therapeutic DLI. CD4 count ≥ 100 /μL at 6–14 months.	Lewalle et al. (46)
AML, ALL, and CML	Unmanipulated graft, *in vivo* T cell depletion with ATG, post-transplant immunosuppression	Children (*n* = 13) Adults (*n* = 5)	0.07–4.4 × 10^8^/kg (median 0.58 × 10^8^/kg); Administered after leukemia relapse; ≥2 doses in five patients	12 patients received CSA or low-dose MTX for 2–4 weeks	aGVHD III-IV in 6/20 patients; 15 patients achieved CR at a median of 289 days after DLI; 2 year DFS: 40%	Huang et al. (47)
High-risk leukemia/lymphoma	Unmanipulated graft, *in vivo* T cell depletion with ATG, post-transplant immunosuppression	Adults (*n* = 31)	0.4–6.9 × 10^7^/kg (median 1.8 × 10^7^/kg); 45–240 (median 77) days after HSCT (prophylactic)	CSA	aGVHD II-IV in 55% and aGVHD III-IV in 10% at 100 days after DLI; severe cGVHD in 18%. TRM 26% and relapse rate 33% at 2 years after DLI	Gao et al. (48)
Malignant and non-malignant diseases	CD34+ selected stem cell graft without post-transplant immunosuppression	Children (*n* = 31) Young adults (*n* = 4)	3–5 × 10^4^/kg; 30–42 days after HSCT (prophylactic); Rituximab given 1 day before DLI in the last 10 patients	MTX	DLI dose of 5 × 10^4^/kg resulted in CD4 count > 100/μL by 120 days in 67% of patients and aGVHD II-IV in 11%; Fatal viral and fungal infections in 11%; 2 year OS: 69% for patients in remission at transplant.	Gilman et al. (49)

*aGVHD, acute graft- vs.-host disease; AML, acute myeloid leukemia; ALL, acute lymphoblastic leukemia; CML, chronic myeloid leukemia; CSA, cyclosporine; DFS, disease-free survival; MDS, myelodysplastic syndrome; MTX, methotrexate; NHL, non-Hodgkin's lymphoma; TRM, treatment-related mortality; OS, overall survival*.

## *In vitro* T Cell Manipulation for Adoptive Cellular Therapy

**Figure 1:**
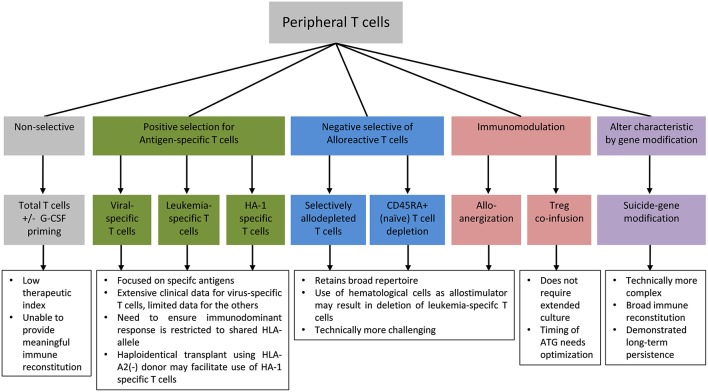
Strategies for adoptive T cell transfer.

The high frequency of alloreactive T cells relative to the frequency of anti-pathogen and anti-leukemic T cells meant that the addback of unmanipulated T cells have a low therapeutic index in haploidentical HSCT and are unlikely to confer meaningful reconstitution of protective immunity without unacceptable risk of severe GVHD. Therefore, *in vitro* T cell engineering approaches to improve safety, reduce alloreactivity, and enhance protective anti-pathogen and anti-leukemic responses following allogeneic HSCT are of particular interest in this setting. Specific approaches to mitigate the risk of GVHD include enrichment for antigen-specific T cells to selectively reconstitute pathogen-specific or leukemia-specific T cells, immunomodulation of alloreactive T cells or co-infusion of suppressor cells, and safety switch gene-modification to enable the conditional deletion of T cells in the event of GVHD or other adverse events (Figure 1).

## Antigen-Specific T cells

### Virus-Specific T Cells

One of the earliest forms of T cell therapy is the adoptive transfer of donor-derived virus-specific T cells, which can be effective in the prevention or treatment of post-transplant CMV infection (50) and EBV-associated post-transplant lymphoproliferative disease (PTLD) (Table 2) (55). The early success of this approach has led to the development of T cells that can target other pathogens, including adenovirus, polyoma viruses, and aspergillus. Repeated rounds of *in vitro* antigenic stimulation to expand virus-specific memory T cells from seropositive donors remains the mainstay for this approach but other technological platforms, including immunomagnetic capture of interferon-γ-producing T cells for rapid infusion has also been successfully applied (52, 56). In the past decade, there has been a strong move from donor-derived T cells toward third-party T cells. Although third-party T cells do not engraft long-term, they are effective and have the benefits of immediate availability, lower cost per treatment and, importantly, are available to patients with seronegative donors, where virus-specific T cells cannot be generated. A full discussion on virus-specific T cells is beyond the scope of this paper and has been reviewed elsewhere (57–59), but it is important to highlight here the implication of HLA-mismatching. In haploidentical HSCT, the dominant virus-specific T cell response in the donor is sometimes restricted to a non-shared HLA, in which case it will not recognize infected recipient cells. This was illustrated by a patient with recipient-derived EBV-associated PTLD after a maternal haploidentical transplant which failed to respond to donor-derived EBV-specific T cells. It was later discovered that the dominant EBV-specific response in the maternal donor was restricted to a non-shared HLA; and subsequent infusion of third-party T cells that had EBV-specific activity restricted to the patient's HLA resulted in a complete response (60).

**Table 2:** Selected studies using virus-specific T cells that enrolled predominantly haploidentical HSCT patients.

**Underlying disease**	**Transplant protocol**	**Study cohort**	**Methodology used to generate virus-specific T cells**	**T cell source**	**T cell dose/time of infusion**	**Outcome**	**References**
AML, ALL, CML, MM, lymphoma	CD34+ selected stem cell graft, myeloablative conditioning, ATG. No post-transplant immunosuppression	Adults (*n* = 25)	Repeated rounds of stimulation with CMV lysate	Stem cell donor	Escalating doses: 10^5^/kg−3 × 10^6^/kg; 13–37 days after HSCT (prophylaxis)	Reduced CMV reactivation compared to control and accelerated pathogen-specific immune reconstitution; aGVHD grade II in 1/25 patients.	Perruccio et al. (51)^*^
Acute leukemia and others	Haploidentical HSCT with post-transplant immunosuppression	Children (*n* = 8) Adults (*n* = 3)	IFN-γ cytokine capture for CMV-pp65 specific T cells	Stem cell donor	2.5–16.6 × 10^3^/kg; in patients with refractory CMV infection	Complete or partial viral clearance in 10/11 patients;No *de novo* GVHD	Feuchtinger et al. (52)^†^
AML/ALL and others	Unmanipulated graft, *in vivo* T cell depletion with ATG, post-transplant immunosuppression	Children and adults (*n* = 32)	Repeated rounds of stimulation with CMV-pp65 peptide mixture	Stem cell donor	0.7–15.4 × 10^7^/kg (median 2.7 × 10^7^/kg); ≥2 doses in 14 patients with refractory CMV infection; 53–127 (median 69) days after HSCT	Viral clearance in 27/32 patients within 4 weeks; aGVHD grade II in 1/32 patients	Pei et al. (53)
Malignant and non-malignant diseases	Haploidentical (*n* = 5) and alternative donor (*n* = 33) HSCT	Children and adults	Multivirus-specific T cell lines	Third-party off-the-shelf	2 × 10^7^ total cells; Treatment of infection with one (*n* = 31) or two (*n* = 7) viruses	Response rates: 94% for CMV (*n* = 17), 100% for BK virus (*n* = 16), 71% for Adenovirus (*n* = 7), 67% for HHV-6 (*n* = 3), 100% for EBV (*n* = 2). aGVHD grade I in 2/38 patients	Tzannou et al. (54)

*aGVHD, acute graft- vs.-host disease; AML, acute myeloid leukemia; ALL, acute lymphoblastic leukemia; CML, chronic myeloid leukemia; HHV-6, Human Herpesvirus 6; MM, multiple myeloma*.

* *This study also included patients who received Aspergillus-specific T cells*.

† *This study also included patients who received matched unrelated or umbilical cord blood transplant*.

### Leukemia-Specific T Cells

Immunogenic proteins that are differentially expressed by leukemic cells and normal hematopoietic cells can be targeted by immunotherapy. In general, the expansion of leukemia-specific T cells is significantly more challenging than that for virus-specific T cells because of their lower precursor frequency and predominance in the naïve T cell compartment, but it can nonetheless be accomplished from both healthy donors and cancer patients. One of the first targets pursued clinically is Wilms tumor protein-1 (WT-1), which is overexpressed in a number of leukemias and solid tumors and encode a range of immunogenic peptide epitopes that can be used to successfully expand WT-1 specific T cells (61–63). Over the years, a number of other leukemia- and tumor-associated antigens have been identified and their epitopes mapped, including PR1 (64), BCR-ABL (65), and PRAME (66). Two early phase small-scale clinical trials have shown the feasibility of using WT-1 specific donor T cells in allogeneic stem cell transplant, and disease response was reported in two patients, one of whom had a prolonged remission (67, 68). Although mapped epitopes provide the first proof-of-principle, it is also feasible to generate antigen-specific T cells from healthy donors using overlapping peptide libraries, which does not require prior epitope-mapping or knowledge of HLA-restriction (69). However, as is the case with virus-specific T cells, HLA-restriction remains a critical consideration in haploidentical transplantation as around half of the T cell response is anticipated to be restricted to non-shared HLA.

### T Cells Targeting Minor Histocompatibility Antigens

Minor histocompatibility antigens are polymorphic peptides that are presented on MHC. In the allogeneic transplant setting, minor histocompatibility antigens are most commonly the result of single nucleotide polymorphism (SNP) that differs between donor and recipients (70). Some of these antigens demonstrate preferential expression on hematopoietic cells, which make them promising targets for immunotherapy after allogeneic transplant. One such candidate is HA-1, the immunogenic form of which is presented by HLA-A2. Around half of the European population carry the immunogenic HA-1 allele (71), and if they are also HLA-A2(+), they may benefit from the adoptive transfer of T cells targeting HA-1. However, it is very difficult to expand HA-1-specifc T cells from healthy donors to clinically relevant numbers because they are present in very low frequencies within the naïve T cell compartment (72). Hence, gene-modification with TCR alpha and beta chains cloned from HA-1-specific T cells has been pursued (72) and is currently undergoing clinical trial (NCT03326921). In HLA-matched setting, the donor would need to be homozygous for the non-immunogenic form of HA-1 in order to avoid fratricide of donor hematopoietic cells; but in haploidentical transplantation, the HA-1 genotype is no longer relevant if an HLA-A2(–) donor is used, thus expanding the donor pool.

## Selective Removal of Alloreactive T Cells

### Selective Allodepletion and Anergy Induction

Although the adoptive transfer of antigen-specific T cells can be highly effective in specific infectious complications, they are highly targeted and do not confer broad protective immunity. A converse approach is the selective depletion or anergy induction of alloreactive T cells which, in theory, will retain broad immune repertoire minus alloreactivity (Table 3). The process involves the co-culture of donor T cells with irradiated recipient blood cells to activate alloreactive donor T cells: in allodepletion, activated T cells are removed based on their expression of activation markers or other properties associated with cell activation (73); and in alloanergy induction, the addition of co-stimulation blockade during co-culture results in the generation of anergic T cells (77, 81). A number of activation markers and depletion strategies have been investigated, two of which have been the focus of clinical trials. In the first method, an immunotoxin comprising a CD25-antibody conjugated to ricin is used to deplete CD25(+) alloreactive T cells *in vitro*. This could effectively reduce alloreactive T cells to <1% of the T cell population and retain responses to pathogens. The infusion of 1–8 × 10^5^/kg allodepleted T cells was found to be safe in haploidentical transplant setting and could help accelerate T cell reconstitution not only numerically but also in diversity, with broad TCR repertoire and evidence of CMV and EBV-specific T cell reconstitution (73, 74). A second method involves photodynamic removal of activated T cells, which have reduced p-glycoprotein-mediated efflux of a photosensitizer, TH9402 (82). Following a phase I dose-finding study demonstrating grade I–II GVHD but no life-threatening grade III–IV GVHD (75), a multicenter phase II study was conducted. Twenty-three patients with high-risk acute leukemia were given photodepleted donor T cell products at a dose of 2 × 10^6^ CD3+ cells/kg at a median of 28 days after T cell deplete haploidentical HSCT: 5 patients developed grade I-II acute GVHD and the rates of leukemia relapse and non-relapse mortality were lower compared to historical controls (76).

**Table 3:** Selected studies using allodepletion, alloanergy induction, and other immune modulation to facilitate T cell addback after haploidentical HSCT.

**Underlying disease**	**Transplant protocol**	**Study cohort**	**Method for generating T cell therapy product**	**T cell dose/time of infusion**	**Outcome**	**References**
Malignant and non-malignant diseases	CD34+ selected graft, myeloablative conditioning, ATG. No post-transplant immunosuppression	Infants or young children (*n* = 15)	Anti-CD25 immunotoxin-mediated allo-depletion using non-donor parent PBMC as alllo-stimulators	1–8 × 10^5^/kg in dose-escalating cohorts; 15–47 days after HSCT	CD4 count ≥200/μL after 13 weeks (median) in 10 evaluable patients; massive expansion of T cells within 4 weeks of T cell infusion in three patients with CMV infection; aGVHD II-IV in 0/15 patients.	Andre-Schmutz et al. (73)^*^
Malignant and non-malignant diseases	CD34+ selected graft, myeloablative and non-myeloablative-conditioning, alemtuzumab. 7/16 patients received tacrolimus/CSA	Children (*n* = 14) Adults (*n* = 2)	Anti-CD25 immunotoxin-mediated allo-depletion using recipient EBV-LCL as allo-stimulators	Two dose levels: Level 1: 10^4^/kg (*n* = 8), Level 2: 10^5^/kg (*n* = 8); Days +30, +60 and +90 after HSCT	Dose level 2 significantly accelerated reconstitution of both CD4 and CD8 T cells, with CMV and EBV-specific responses observed in 4 of 6 evaluable patients at 2–4 months after HSCT; aGVHD II-IV in 2/16 patients	Amrolia et al. (74)
Malignant diseases	CD34+ selected graft, myeloablative conditioning, ATG. No post-transplant immunosuppression	Adults (*n* = 19)	Photodepletion with TH9402 using recipient PBMC as allo-stimulators	Phase I dose-finding study: 1 × 10^4^/kg−5 × 10^6^/kg; 28–40 days after HSCT	aGVHD I–II in 5/19 patients No aGVHD III–IV	Roy et al. (75)
High-risk AML and ALL	CD34+ selected graft, myeloablative conditioning, ATG.	Adults (*n* = 23)	Photodepletion with TH9402 using recipient PBMC as allo-stimulators	Phase II study: 2 × 10^6^/kg; 28 days (median) after HSCT	aGVHD I–II in 22 %; 1 year TRM: 32%	Roy et al. (76) (Abstract)
Malignant and non-malignant diseases	Allo-anergized bone marrow with post-transplant CSA/MTX	Children (*n* = 9) Young adults (*n* = 3)	CTLA-4-Ig mediated alloanergy induction, using recipient PBMC as allo-stimulators	1.6–5.5 × 10^7^/kg (contained in BM graft) on Day 0 of HSCT	CD4 count ≥400/μL by 6 months and CD4/CD8 ratio ≥ 1.4 by 7 months in all five surviving patients; Gut aGVHD in 3/11 patients; DFS at last follow-up: 5/12 patients	Guinan et al. (77)
High-risk acute leukemia or MDS	CD34+ selected graft, myeloablative conditioning, ATG. No post-transplant immunosuppression	Children (*n* = 5) Adults (*n* = 11)	Anti-B7.1-mediated alloanergy induction using PBMC from recipient or a second haploidentical donor as allo-stimulators	Escalating dose levels: Level 1: 10^3^/kg, Level 2: 10^4^/kg, Level 3: 10^5^/kg; 35–42 days after HSCT	Functional virus-specific CD4 T cells detectable at a median of 9, 3, and 2.5 months and CD8 T cells at median of 9, 4, and 3 months in dose level 1/no DLI, dose level 2, and dose level 3, respectively; aGVHD II-IV in 5/16 patients; DFS at last follow-up: 4/16 patients	Davies et al. (78)
High-risk AML, ALL, lymphoma	CD34+ selected graft, myeloablative conditioning. No ATG or other serotherapy. No post-transplant immunosuppression.	Adults (*n* = 26)	Fresh Tregs (G-CSF mobilized) isolated by CD25-immuno-magnetic selection on Day (– 4), CD34+ selected stem cells and Tcon on Day 0.	Two dose levels: Level 1: Treg 2 × 10^6^/kg + Tcon 0.5–1 × 10^6^/kg (*n* = 21); Level 2: Treg 4 × 10^6^/kg + Tcon 2 × 10^6^/kg (*n* = 5)	CD4 count ≥100/μL and ≥ 200/μL at median of 42 (28–135) and 67 (40–146) days after HSCT, respectively; CD8 count ≥100/μL and ≥200/μL at median of 38 (19–95) and 48 (21–95) days, respectively; aGVHD II-IV in 2/26 patients; 12-month DFS: 46%	Di Ianni et al. (79)
High-risk AML and ALL	CD34+ selected graft, myeloablative conditioning. No serotherapy (*n* = 25), ATG or alemtuzumab (*n* = 18). No post-transplant immunosuppression.	Adults (*n* = 43)	As above	Treg 2.5 × 10^6^/kg (mean) + Tcon 1.1 × 10^6^/kg (mean)	CD4 count ≥100/μL and ≥200/μL at median of 40 (25–150) and 55 (45–160) days after HSCT, respectively; CD8 count ≥100/μL and ≥200/μL at median of 45 (18–100) and 60 (50–140) days, respectively; aGVHD II-IV in 15%; Relapse: 2/41 patients; 46-month DFS: 56%	Martelli et al. (17)^†^
AML, ALL or lymphoma	CD34+ selected graft, myeloablative conditioning. ATG.	Adults (*n* = 12)	IL-10 mediated alloanergy induction using recipient CD3-depleted peripheral blood cells as allo-stimulators	T cells dose of 3–5 × 10^5^/kg; 28–64 days after HSCT	CD4 count >150/μL and CD8 count >100/μL at a median of 30 (15–102) days in the four long-term survivors; aGVHD II-III in 5/12 patients; DFS at last follow-up: 4/12 patients	Bacchetta et al. (80)

*aGVHD, acute graft- vs.-host disease; AML, acute myeloid leukemia; ALL, acute lymphoblastic leukemia; BM, bone marrow; CSA, cyclosporine; CTLA-4-Ig, cytotoxic T-lymphocyte-associated protein-4-immunoglobulin bound to human IgG1 constant region; DFS, disease-free survival; MDS, myelodysplastic syndrome; MM, multiple myeloma; MTX, methotrexate; OS, overall survival; PBMC, peripheral blood mononuclear cells; TRM, transplant related mortality*.

* *This study included haploidentical HSCT (n = 13) and matched unrelated HSCT (n = 3), with one patient receiving two transplants*.

† *Some of the patients (n = 24) in this study have been reported in the Di Ianni study (79)*.

The induction of anergy in alloreactive donor T cells can be achieved by blocking B7/CD28 costimulation during co-culture. This can be achieved by adding CTLA-4-Ig, which is a soluble fusion protein of CTLA-4 extracellular domain to human IgG1 constant region (77), or anti-B7-1 and B7-2 antibodies during co-culture (18). This process can reduce the precursor frequency of alloreactive T cells by 1–4 logs, as measured by IL-2 production in one-way mixed lymphocyte response co-culture with irradiated recipient cells. Seminal works have demonstrated that when used with routine post-transplant immunosuppression, haploidentical alloenergized bone marrow grafts could successfully engraft, rates of infection were low, and there were no GVHD-associated deaths (77). More recently, alloanergized T cells were used as DLI at 35–42 days after CD34-selected haploidentical HSCT; 16 patients were treated: low dose DLI (10^3^ T cells/kg; *n* = 4) did not result in acute GVHD but also had little impact on T cell reconstitution, whereas higher doses (10^4^-10^5^ T cells/kg; *n* = 12) significantly accelerated T cell recovery, although five patients developed grade II–IV acute GVHD. Interestingly, *in vitro* alloanergy induction was found to expand CD4^+^CD25^+^CD127^low^ Tregs within the graft but this did not impair the expansion of antigen-specific T cells *in vivo*, with patients on higher dose levels demonstrating reconstitution of adenovirus-, CMV-, and WT-1-specific T cells (78).

These early phase proof-of-concept studies on the ability of allodepletion and alloanergy induction in promoting engraftment and T cell immune reconstitution with clinically acceptable rates of GVHD are highly promising and call for further studies. One of the most critical considerations in this field is the source of recipient antigen-presenting cells, which can be limiting in heavily pre-treated leukopenic patients. This can be overcome by the use of EBV-transformed lymphoblastoid cell lines (EBV-LCL), which can be generated from small numbers of B cells and expand into large numbers (74), or the use of a second haploidentical family member as the source of stimulator cells (78). However, more difficult to overcome is the reliance on hematopoietic cells as stimulators, which may have the unwanted effect of depletion or anergization of leukemia-specific and hematopoietic-restricted minor histocompatibility antigen-specific T cells, thus reducing their graft- vs.-leukemia effect; and at the same time, the retention of tissue-specific alloreactive T cells that can mediate GVHD.

### Naïve T Cell Depletion

T cells that mediate GVHD largely reside within the naïve T cell compartment (83), whereas virus-specific T cells largely reside within the memory T cell compartment. In the past few years, the immunomagnetic depletion of CD45RA(+) naïve T cells has emerged as an elegant and relatively simple method to deplete alloreactive T cells whilst retaining virus-specific responses (84, 85). In the haploidentical setting, 17 high-risk patients received T cell depleted grafts with the addition of CD45RA-depleted T cell fraction, which contained <10^3^/kg CD3^+^CD45RA^+^ T cells and a median of 10^8^/kg CD45RA(-) T cells (86): there was rapid reconstitution of memory T cells and remarkably, none of the patients developed acute GVHD. This promising approach is now undergoing further investigation (87) with several clinical trials in progress in the haploidentical setting (NCT02960646; NCT03849651; NCT02790515).

## Co-infusion of Regulatory T Cell Subsets

Tregs are CD25^+^Foxp3^+^ CD4 T cells, which are the key mediators of peripheral tolerance. Their ability to prevent and attenuate GVHD is well-established in preclinical studies and clinical correlative studies (32, 88, 89). Adoptively transferred Tregs can reduce the risk of GVHD associated with the add-back of Tcons (Table 3). In this approach, Tregs were isolated by CD25 immunomagnetic selection and 2 × 10^6^ /kg Tregs were infused 4 days prior to the infusion of stem cell graft, which was given together with controlled numbers (0.5–2 × 10^6^/kg) of Tcons, without any post-transplant immunosuppression (17, 79). This approach was shown to accelerate CD4 and CD8 T cell immune reconstitution, with low rates (15%) of acute GVHD grade ≥2, and significant improvement in clinical outcome compared to historical controls. T cells specific to CMV, adenovirus, Aspergillus and other pathogens were detectable at much earlier timepoints compared to historical controls; and although infection remained a significant challenge, there were significant improvements in the rates of CMV reactivation and there were no target organ CMV disease (79). However, Tregs constitute only 5–10% of total CD4 T cells and it is often challenging to isolate Tregs in sufficient numbers and purity. Tregs can be expanded *in vitro* by thousands-fold without loss of purity and suppressive function (90, 91). Early phase clinical trials have shown that *in vitro* expanded Tregs are safe in cord blood transplantation, but their impact on GVHD is difficult to assess (92, 93) and this approach has not been reported in the haploidentical setting.

*In vitro* induced Tregs (iTregs) can be generated by the activation of conventional CD4 T cells in the presence of transforming growth factor-β (TGF-β) and rapamycin. Although iTregs have suppressive abilities *in vitro*, their suppression of GVHD in preclinical models requires the administration of rapamycin, without which they revert to pathogenic conventional T cells (92, 94). Type-1 regulatory T cells (Tr1 cells) is a subtype of Foxp3(–) CD4 T cells with regulatory function. They can regulate alloantigen-specific immune response via granzyme B-mediated killing of myeloid antigen-presenting cells and the production of immunomodulatory cytokines, chiefly interleukin-10 (IL-10) and TGF-β (95, 96). In preclinical models, the adoptive transfer of Tr1 cells is effective in suppressing GVHD (96). In a proof-of-concept clinical study, Tr1 cells were generated by co-culture of donor peripheral blood mononuclear cells with recipient CD3-depleted cells in the presence of IL-10 (Table 3). Twelve patients received delayed add-back of Tr1 cells after CD34-selected haploidentical HSCT: 7 died before day 100, the remaining 5 had accelerated immune reconstitution, but all had acute GVHD grade II–III (80). Thus, the results for these alternative regulatory T cell populations are mixed and naturally occurring Tregs which are biologically well-defined remain the most established form of immunomodulatory T cell population at present.

## Safety Switch Gene-Modified T Cells

All T cell add-back strategies carry a risk of life-threatening GVHD. Although the risk is dose-related, it is not predictable for a given individual, and a cell dose that is safe for all is ineffective in haploidentical transplantation where there is a narrow therapeutic window. Safety switches, also known as suicide genes, refers to gene modification that enables the conditional elimination of infused cells and all their progenies in the setting of adverse events. This technology has potential application in a broad range of cellular therapeutics but their proof-of-concept was in allogeneic HSCT where the safety switch can be triggered and donor T cells deleted in the event of life-threatening GVHD (Table 4).

**Table 4:** Clinical trials using safety switch gene-modified T cells after haploidentical HSCT.

**Underlying disease**	**Study/Transplant protocol**	**Study cohort**	**Method for generating T cell therapy product**	**T cell dose/time of infusion**	**Outcome**	**References**
High-risk leukemia	CD34+ selected stem cell graft, myeloablative conditioning, ATG. No post-transplant immunosuppression	Adults (*n* = 28)	HSVtk modification of T cells	Intrapatient dose escalation at monthly interval if no GVHD: Dose level 1: 10^6^/kg, Dose level 2: 10^7^/kg, Dose level 3: 10^6^/kg + IL-2, Dose level 4: 10^7^/kg + IL-2; Starting 28 days after HSCT	HSVtk T cells engrafted in 22 patients: CD3+ count >100/μL at median of 75 (34–127) days after HSCT and 23 (13–42) days after HSVtk T cell infusion; aGVHD II-IV in 9/28, extensive cGVHD in 1/28, all resolved with ganciclovir administration	Ciceri et al. (97) Oliveira et al. (98)
High risk acute leukemia and MDS	Phase I, allodepleted T cell add-back after CD34-selected transplant. 9/10 patients received alemtuzumab for *in vivo* T cell depletion (NCT00710892)	Children (*n* = 10)	iCasp9 modification with prior T cell allodepletion using CD25-immunotoxin	Escalating doses: Dose level 1: 10^6^/kg Dose level 2: 3 × 10^6^/kg Dose level 3: 10^7^/kg; 1st dose: 30–124 days after HSCT (4 patients received 2nd dose)	iCasp9 T cells: 54/μL and 63/μL at 1 and 2 years after HSCT, respectively; aGVHD I- II in 4/10 patients, all resolved with AP1903 administration	Di Stasi et al. (16) Zhou et al. (99)
Acute leukemia and other malignant diseases	Phase I, non-allodepleted T cell add-back after CD34-selected transplant. All 10 patients received alemtuzumab for *in vivo* T cell depletion (NCT01494103)	Children (*n* = 9) Adults (*n* = 3)	iCasp9 modification of T cells	Dose level 1: 10^4^/kg (*n* = 5), Dose level 2: 5 × 10^5^/kg (*n* = 2, one patient received 2nd dose), Dose level 3: 10^6^/kg (*n* = 2), Dose level 4: 5 × 10^6^/kg (*n* = 3); 1st dose: 31–82 days after HSCT	Viral-specific iCasp9 T cells were detected in eight patients; aGVHD I–II in 3/12, all resolved after AP1903 administration	Zhou et al. (100)
Acute leukemia and non-malignant diseases	Pilot followed by phase II study. TCR αβ+ T cell and CD19+ B cell depleted stem cell graft, myeloablative conditioning and ATG. No post-transplant immunosuppression NCT02065869 and EudraCT:2014-000584-41 Long term follow-up: NCT03733249 and EudraCT: 2016-003226-16	Children (*n* = 108)	iCasp9 modification of T cells (BPX-501)	Pilot (*n =* 9): dose escalation: 2.5 × 10^5^/kg, 5 × 10^5^/kg, 10^6^/kg; Phase II: 1 × 10^6^/kg (n = 99); All on Day 0 of transplant	iCasp9 (BPX-501) T cells peaked at 9 months after infusion (mean 144/μL) and persisted for at least 2 years (mean 62/ μL); Expansion of iCasp9 T cells correlates with CMV reactivation.	Merli et al. (101) (Abstract)
High-risk acute leukemia	CD34-selected stem cell graft with myeloablative conditioning and ATG for *in vivo* T cell depletion (ACTRN12614000290695)	Adults (*n* = 3)	iCasp9 modification of T cells	Dose level 1: 0.5 × 10^6^/kg (*n* = 2), Dose level 2: 1 × 10^6^/kg (*n* = 1) Additional doses allowed; 1st dose at 25–26 days after HSCT	One patient in DFS at >3.5 years; One patient died of relapse; One patient died of GVHD	Zhang et al. (102, 103)

*aGVHD, acute graft- vs.-host disease; DFS, disease free survival; MDS, myelodysplastic syndrome*.

### Safety Switch Technologies

The first clinically tested safety switch was herpes simplex virus thymidine kinase (HSVtk). This kinase catalyzes the monophosphorylation of ganciclovir and related nucleoside analogs, which is then converted by cellular kinases to di- and tri-phosphates, leading to arrest of DNA synthesis and subsequent cell death. T cells transduced with HSVtk retained their ability to mediate protective immunity *in vivo* and, in patients who developed GVHD, the HSVtk T cells could be eliminated by ganciclovir, with resolution of GVHD (104, 105). This strategy thus allows the administration of T cells with broad specificity and in numbers sufficient for mediating protective immunity. However, HSVtk as a safety switch has a number of drawbacks: the mechanism is dependent on cell cycle, thus killing can be delayed and is limited to proliferating cells; it precludes the use of ganciclovir and acyclovir as anti-virals; and it is a foreign protein which can elicit CD4 and CD8 immune response (106), although this is not universal and long-term persistence of HSVtk T cells has been reported (98).

The past decade has seen the development and clinical validation of inducible caspase 9 (iCasp9) as a safety switch. This technology is based on a cell membrane-permeable small molecule dimerizing drug, AP1903 (also known as Rimiducid), which binds with very high affinity and specificity to an engineered drug-binding domain. The drug-binding domain is derived from human FKBP12, with a single amino acid substitution from phenylalanine to valine (FKBP12-F36V) (107, 108). The iCasp9 transgene consists of FKBP12-F36V, joined via a short flexible linker to human caspase 9, without the caspase activation and recruitment domain (CARD), which is now superfluous (107). The administration of AP1903 induces dimerization of caspase 9, which activates the terminal effector caspase, caspase 3, with rapid induction of apoptosis. This system has a number of benefits over HSVtk: it is almost fully human-derived and hence much less likely to be immunogenic, it does not preclude the use of anti-virals and, importantly, the mechanism of cell death is cell-cycle independent, with >90% cell death within 30 min *in vitro* and *in vivo* (16, 107).

A couple of other safety switches based on cell surface expression of epitopes that enables their elimination by clinical monoclonal antibodies have also entered clinical trial, mainly in the area of chimeric antigen receptor (CAR) T cell therapy rather than in allogeneic HSCT. RQR8 is a relatively small transgene that encodes two epitopes: one from CD34, which binds to a clinical grade CD34 antibody for immunomagnetic selection; and a CD20 epitope, which functions as a safety switch in conjunction with rituximab, a chimeric antibody which mediates antibody-dependent cytotoxicity widely used in the treatment of CD20-positive B cell malignancies (109). Another strategy is to express a truncated form of human epidermal growth factor receptor (EGFR), which enables the cells to be deleted by cetuximab, a chimeric antibody used to treat EGFR-expressing colorectal and head and neck cancer (110). Both RQR8 and truncated EGFR have been incorporated within other forms of gene therapy but there has not been a need to activate the safety switch and hence their clinical efficacy *in vivo* is yet to be demonstrated. These technologies have not been used as standalone safety switch in post-transplant T cell addback.

### Clinical Experience of Safety Switch Modified T Cells in Haploidentical Transplantation

HSVtk has undergone clinical trial in HLA-matched sibling allogeneic HSCT (105, 111) and haploidentical HSCT (97). In a phase I–II multicentre study in haploidentical HSCT, patients received 10^6^/kg−10^7^/kg HSVtk T cells starting 28 days after myeloablative transplantation using CD34-selected stem cell graft and *in vivo* T cell depletion with ATG, without any post-transplant immunosuppression (97). Additional doses were allowed at monthly intervals in the absence of GVHD. Of the 50 enrolled patients, 28 were eligible to receive HSVtk T cells, none of whom had detectable T cells prior to HSVtk infusion. Twenty-two patients achieved T cell count >100 cells/μL within 13–42 days (median 23) after HSVtk T cell infusion, and reconstitution of EBV and CMV-specific T cells was observed. Ten patients developed acute GVHD and one developed chronic GVHD, all of which were controlled by the infusion of ganciclovir.

The iCasp9 safety switch was first tested in the haploidentical HSCT setting using donor T cells that were first allodepleted before iCasp9 transduction (112). Ten patients received 10^5^-10^7^ iCasp9 T cells/kg between day 30–124 after CD34-selected stem cell transplant. The iCasp9 T cells were found to engraft, expand, contribute to the reconstitution of both CD4 and CD8 T cells, and confer anti-viral immunity (16, 99). Four patients developed acute GVHD and received AP1903, which eliminated >90% of iCasp9 T cells within 30 min, with a further 0.5 log reduction in the subsequent 24 h and resolution of GVHD within 24–48 h (16). The safety of this approach led to a second clinical study using non-allodepleted iCasp9 T cells at 1 × 10^4^–5 × 10^6^ cells/kg (100). Again, immune reconstitution was accelerated and 4 patients developed GVHD, all of which were successfully managed with AP1903 administration. The largest study using iCasp9 T cells to date is a phase II multicenter study conducted in Italy on children who have undergone TCRαβ- and B cell-deplete haploidentical HSCT (NCT02065869). In a preliminary report, 108 patients received 0.25–1 × 10^6^ iCasp9 T cells within 1 month of transplantation and it was shown that the iCasp9 T cells engrafted, peaked at 9 months after infusion and persisted for at least 2 years (101). Encouraged by these clinical successes, larger multicenter studies are underway in the USA and Europe (Table 5).

**Table 5:** Additional clinical trials on ClinicalTrials.gov involving iCasp9 T cell addback following haploidentical HSCT^*^.

**Registration ID**	**Underlying disease**	**Study protocol**	**Study cohort**	**Study Locations**	**Posted Date/status**	**Comment**
NCT03301168	Malignant and non-malignant diseases	Phase II, T cell add-back after TCR αβ, and B cell deplete stem cell transplant	Children	Multiple locations, USA	Oct-4-2017 Active/not recruiting	
NCT01744223	Malignant diseases	Phase I/II, T cell add-back after T cell deplete transplant	Adults	Multiple locations, USA	Dec-6-2012 Active/not recruiting	
NCT02477878; EudraCT:2015-005176-17	Malignant diseases	Phase I, treatment of relapse or minimal residual disease after allogeneic HSCT	Adults	Multiple locations, USA & Italy	Jun-23-2015 Active/not recruiting	This study includes matched related and haploidentical HSCT
NCT03459170	Malignant diseases	Phase I, treatment of relapse or minimal residual disease after allogeneic HSCT	Children	Italy	Mar-8-2018 Recruiting	This study includes matched related and haploidentical HSCT
NCT03639844	Non-malignant diseases	T cell add-back after TCR αβ and B cell deplete transplant	Children and young adults	Multiple locations, USA	Aug-21-2018	Expanded access protocol
NCT03699475	Malignant diseases	Phase II/III, TCR αβ and B cell deplete transplant with iCasp9 T cell addback vs. Haploidentical HSCT with PTCy	Children and adults	Nashville, TN and San Antonio, TX	Oct-8-2018/Recruiting	
NCT02231710	Non-malignant diseases	Phase I, T cell add-back after T cell deplete transplant	Children and adults	Seattle, WA	Sep-4-2014 Active/not recruiting	Closed after enrolling one patient

* *Excludes studies listed on Table 4*.

The addback of safety switch gene-modified T cells does not inhibit endogenous T cell reconstitution. Indeed, the infusion of HSVtk was associated with an increase in circulating TCR excision circles (TREC) and CD31^+^ recent thymic emigrants, and an expansion of thymic tissue, which seemed to coincide with a peak in serum IL-7 level (113). Similarly, an increase in endogenous naïve T cell numbers after the infusion of iCasp9 T cells has also been reported (99). Together, these findings suggest that the transfer of safety switch gene-modified T cells can promote thymic output; but this phenomenon requires confirmation and further study.

### Fate of Safety Switch Gene-Modified T Cells

Safety switch gene-modified T cells can be tracked long-term because the transgene is integrated within the cell genome and passed on to all daughter cells. The transduced T cells can be identified by PCR and, in many cases, also by flow cytometry for surface markers contained within the transgene. For example, HSVtk gene-modified T cells co-express ΔLNGFR and iCasp9 T cells co-express ΔCD19, which enable them to be readily distinguished on flow cytometry from T cells contained within the stem cell graft and new thymic emigrants. Using a combination of these techniques, safety switch gene-modified T cells have been shown to persist long-term: iCasp9 T cells can persist for at least 2–4 years (99, 101, 102), and long-term follow-up studies have demonstrated the presence of HSVtk T cells in all memory and effector T cell compartments for up to 14 years after infusion (98).

**Figure 2:**
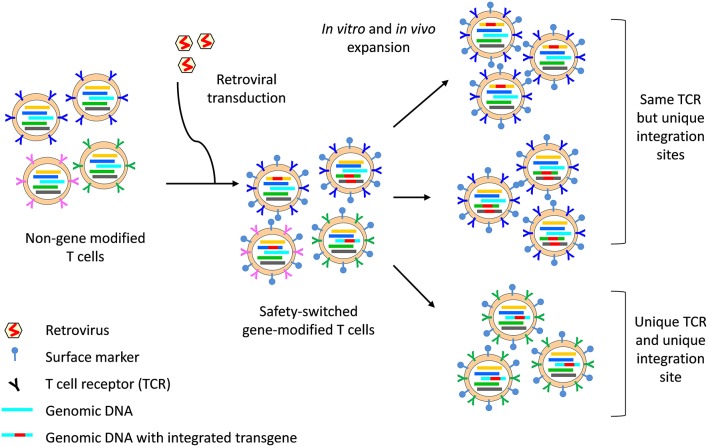
Integration site and TCR analysis on safety switch gene-modified T cells. The safety switch transgene is integrated into the host cell genome, with 1–3 copies of integrants per cell. The region of the genomic DNA in which the transgene integrates (indicated by a unique color) is unique to each integration event and all progenies from that cell can be identified through the unique integration site. Similarly, each T cell bears a unique TCR (indicated by a unique color), which is passed on to all progenies. However, two cells bearing the same TCR may be separately gene-modified, resulting in two clones of T cells which bear the same TCR but with different integration site. Surface markers, for example ΔCD19 or ΔLNGFR, enable identification of safety switch gene-modified T cells by flow cytometry and can be used for cell sort before TCR sequencing. The concurrent analysis of TCR and integration site can provide high resolution data on the clonal origin of safety switch gene-modified T cells.

The clonal origins of safety-switch modified T cells can be tracked with high resolution by TCR analysis and transgene integration site analysis. The integration site refers to the position within the host cell genome in which the transgene has been inserted. Each transduction event results in the integration of the transgene into unique positions within the host cell genome; hence, analysis of transgene integration sites enables the identification of cells that are clonally related and provides information on the source, fate and proliferative capacity of HSVtk and iCaps9 T cells (Figure 2). In the HSVtk studies, there was a high level of TCR diversity in the first few months after adoptive transfer (16), but dominant clonotypes emerged over time (98). TCR and viral integration site analysis showed that these dominant clonotypes preferentially originated from stem cell memory (T_scm_) and central memory (T_cm_) in the infused cell product; and tracking of CMV- and Flu-specific HSVtk T cells showed that antigen exposure was a major driver of *in vivo* expansion and long-term persistence (98). Despite prior *in vitro* expansion and gene modification, safety switch gene-modified T cells retain massive proliferative capacity in response to antigen stimulation: we have shown that a single clone of iCasp9 T cell, bearing the same TCR and viral integration site, could expand 6-log fold in the context of EBV-associated PTLD and contract following resolution of EBV (102).

An interesting feature of iCasp9 safety switch system is that treatment with AP1903 preferentially deletes alloreactive T cells, and the residual iCasp9 T cells retained anti-viral specificity and could subsequently re-expand without causing GVHD (16, 99). Similarly, the kinetics of peripheral blood HSVtk was not significantly different in patients who received ganciclovir vs. those who did not, suggesting a similar phenomenon also operates in the HSVtk system (98). This preferential deletion of alloreactive T cells is in part attributed to higher level of transgene expression in activated T cells, which increase their susceptibility to safety switch activation (112, 114), whereas non-activated viral-specific T cells were relatively spared.

## Timing of Adoptive T Cell Therapy

Adoptive T cell therapy should ideally occur as early as possible to confer protective immunity but, as explained earlier, the cytokine storm in the first 2 weeks post-transplant promotes the priming and Th1/Th17 polarization of alloreactive T cells within the cell product, thus increasingly the risk of GVHD. Furthermore, ATG or, less commonly, alemtuzumab, used during conditioning have long half-life and can eliminate the adoptively transferred T cell product if infused too early. *In vivo* T cell depletion with ATG is critical for the engraftment of haploidentical T cell deplete grafts (9, 35). It is also a standard component of the Beijing approach (11). It is not part of the PTCy approach but the addition of ATG to PTCy is undergoing clinical trial as a means to reduce the rate of GVHD (NCT03689465; NCT03608059; and NCT03367546) (115, 116). The half-life of rabbit ATG (thymoglobulin) is ~6 days (103, 117) and most investigators wait 4–5 half-lives before T cell transfer. The half-life of alemtuzumab is around 8 days and at a standard dose of 100 mg, it will take 56 days (seven half-lives) to fall below the commonly accepted lympholytic level (118).

One approach to enable earlier T cell addback is to administer serotherapy very early, for example, the Treg study administered ATG or alemtuzumab 21 days before transplant (17). Another strategy was to use plasmapheresis prior to T cell addback: a 1–1.5 plasma volume pheresis can half the level of residual ATG (103); however, the relationship between ATG level and *in vitro* cytotoxicity is log-linear, hence halving the ATG level may have only modest biological impact and further studies to define the clinically relevant level of current ATG preparations are required.

## Conclusions

Haploidentical HSCT is now widely accepted as a transplant option for patients who do not have matched sibling donors. It is particularly suitable for adoptive cellular therapy because the donor is readily available for additional donation and it is very feasible to generate advanced cellular therapeutics: sample availability, timing, and the consent process are all less of a barrier compared to using volunteer unrelated donors. In some cases, haploidentical HSCT may be preferable over HLA-matched donor if T cells targeting minor histocompatibility antigen is considered; although this is currently restricted to HA-1 (72), if successful, other hematopoietic-restricted antigens could be identified and similarly targeted (119, 120). On the other hand, it is important to consider shared vs. non-shared HLA-allele in selecting antigen-specific T cell addback after haploidentical HSCT.

The choice of T cell add-back strategy is highly dependent on local expertise and transplant platform. Proof-of-concept studies that were conducted in lymphopenic *ex vivo* T cell deplete settings may not be directly translatable to the non-lymphopenic T cell replete transplant settings. In *ex vivo* T cell deplete transplantation, T cell reconstitution is globally delayed and adoptively transferred T cells can proliferate robustly in a lymphopenic environment; hence, the most effective strategies are likely those that can reconstitute broad protective immunity, such as the infusion of allodepleted or alloanergized T cells, safety-switch gene-modified T cells, and co-infusion of Tcons with Tregs. In T cell replete transplant settings, infection is less of an issue but relapse remains a significant challenge, and strategies that are directed at relapse prevention, such as the use of leukemia-specific T cells and minor histocompatibility antigen-specific T cells may be more relevant. Rapid advances in the broader field of cellular immunotherapy will expand the armamentarium, which will likely incorporate chimeric antigen receptor T cells, off-the-shelf products and NK cell-directed therapy, all of which will help reconstitute protective immunity with an increasingly higher level of safety and efficacy after haploidentical HSCT.

## Author Contributions

S-KT conceived the manuscript. PZ and S-KT reviewed the literature and wrote the manuscript.

### Conflict of Interest Statement

The authors declare that the research was conducted in the absence of any commercial or financial relationships that could be construed as a potential conflict of interest.
